# Evaluation of the sensitivity of a measles diagnostic real-time RT-PCR assay incorporating recently observed priming mismatch variants, 2024

**DOI:** 10.2807/1560-7917.ES.2024.29.28.2400410

**Published:** 2024-07-11

**Authors:** Andrew S Beck, Elena N Lopareva, Hyun Hwang, Derek Hart, Marcos de Almeida, Raydel Anderson, Paul A Rota, Bettina Bankamp

**Affiliations:** 1 Viral Vaccine Preventable Diseases Branch, Division of Viral Diseases, National Center for Immunization and Respiratory Disease, Centers for Disease Control and Prevention, Atlanta, United States; 2 Oak Ridge Institute for Science and Education (ORISE), Oak Ridge, United States; 3 ASRT Inc, Atlanta, United States; 4 Strategic Innovative Solutions Inc., Clearwater, United States

**Keywords:** Measles, Measles Virus, Paramyxovirus, RT-PCR, rRT-PCR, Real-Time PCR, Primer, Variant, N450

## Abstract

We investigated a variant of measles virus that encodes three mismatches to the reverse priming site for a widely used diagnostic real-time RT-PCR assay; reduction of sensitivity was hypothesised. We examined performance of the assay in context of the variant using in silico data, synthetic RNA templates and clinical specimens. Sensitivity was reduced observed at low copy numbers for templates encoding the variant sequence. We designed and tested an alternate priming strategy, rescuing the sensitivity of the assay.

Real-time reverse transcription PCR (RT-rPCR) is widely used to confirm the presence of measles virus (MeV) RNA in clinical specimens. Characteristics are discussed here for the single-target RT-rPCR assay design [1] used at United States (US) Centers for Disease Control and Prevention (CDC) and elsewhere for MeV surveillance and case confirmation (‘CDC assay’ here and in [2]). Performance of the CDC assay is of considerable public health importance, as the test is used widely across surveillance networks including state public health laboratories in the United States and numerous National and Regional Reference Laboratories of the World Health Organization (WHO) Global Measles and Rubella Laboratory Network. Recently, Pérez-Rodríguez et al. reported a MeV sequence variant observed in molecular surveillance data containing three uracil–cytosine nucleotide substitutions (3UC) in the reverse primer binding site of the CDC assay [2]. The authors performed an abbreviated dilution-extinction experiment comparing assay results for a clinical specimen containing the 3UC pattern against a specimen with sequences matching the assay primers, concluding that there was some reduction of assay sensitivity for templates with the 3UC substitution pattern [2,3]. 

Here we examine the presence of 3UC in MeV molecular surveillance data of record and measure changes in the sensitivity for the CDC assay that may result from 3UC variant priming. A redesigned priming strategy is tested against synthetic RNA templates and clinical specimens from CDC archives.

## Priming mismatch variants in molecular surveillance data 

**Figure 1:**
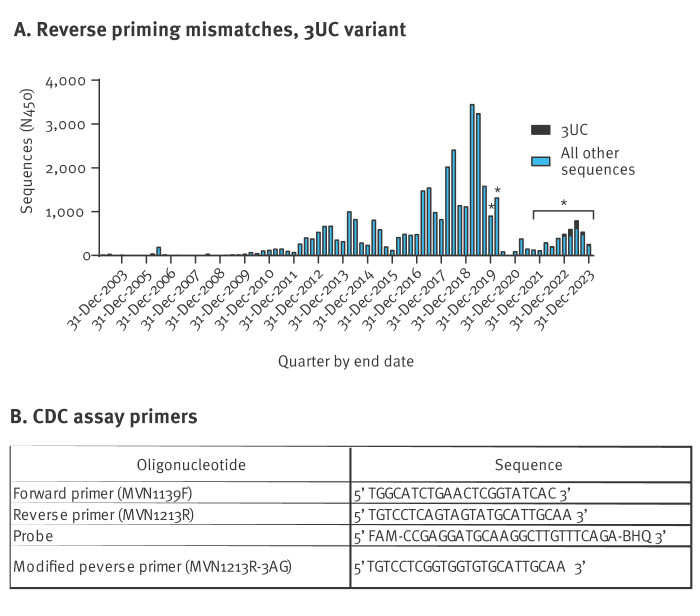
Measles virus RT-rPCR assay primer/probe sequences and 3UC variant surveillance data, 2003–2023 (n = 37,995)

We performed in silico analyses to estimate the prevalence of 3UC and other priming mismatch patterns. Molecular surveillance data for MeV mostly contain sequences generated by the standard genotyping protocol: the 450 nt coding for the C-terminal 150 amino acids of the viral nucleoprotein (N450). The RT-rPCR amplicon falls within this N450 fragment and thus typing sequences may also be used to evaluate RT-rPCR priming accuracy. We obtained a dataset containing all (n = 37,995) MeV genotype B3 and D8 sequences deposited to the WHO global measles virus nucleotide surveillance (MeaNS) database between 2003 and 2023 [4]. The only measles genotypes that had been reported to MeaNS database since 2020 were B3 and D8 indicating that they were the prevalent genotypes worldwide. We extracted unique sequences from the complete set of individual submissions using distinct sequence identifiers (DSId) as previously assigned by the MeaNS database; the unique N450 sequences (n = 2,901) were scanned for mismatches against CDC assay primers and probe using Needleman–Wunsch alignments as implemented in Biopython v1.83 [5]. We identified 32 DSIds containing any 3-mismatch pattern in the reverse primer (MVN1213R) binding region (Table 1); 31 of these DSIds contained the 3UC pattern. The occurrence of 3UC in MeaNS submissions began in the last quarter of 2019 (Figure 1A). Forward priming mismatch and probe mismatch data are summarised in the Supplement.

CDC: Centers for Disease Control and Prevention.

Panel A: Distinct typing sequences (N450) were scanned for reverse priming mismatches; reverse priming variant 3UC started occurring in MeaNS surveillance data in the final quarter of 2019 (*) and then consistently again as a minority of submissions from the final quarter of 2021 (* and bracket).

Panel B: An example table showing CDC assay oligonucleotide sequences along with redesigned reverse primer MVN1213R-3AG.

**Table 1:** Prevalence of CDC MeV RT-rPCR assay priming mismatches in MeaNS N450 submissions, 2003–2023

Mismatches	Forward primer		Reverse primer		Probe	
	n	%	n	%	n	%
DSId (n = 2,901)						
0	2,474	85.28	2,641	91.04	2,490	85.83
1	393	13.55	219	7.55	385	13.27
2	27	0.93	9	0.31	25	0.86
3	3	0.1	32	1.1	1	0.03
4	2	0.07	0	0	0	0
5	2	0.07	0	0	0	0
N450 sequences submitted to MeaNS (n = 37,995)						
0	35,987	94.72	35,966	94.66	35,202	92.65
1	1,921	5.06	1,546	4.07	2,738	7.21
2	80	0.21	16	0.04	54	0.14
3	3	0.01	467	1.23	1	< 0.01
4	2	0.01	0	0	0	0
5	2	0.01	0	0	0	0

CDC: Centers for Disease Control and Prevention; DSId: distinct sequence identifiers; MeaNS: measles virus nucleotide surveillance database; MeV: measles virus.

Mismatch counts were obtained by pairwise sequence alignment. For both unique DSIds and submitted sequences, considerable stability at primer/probe binding regions was observed along the period investigated.

## Assay sensitivity using synthetic RNA templates

**Figure 2:**
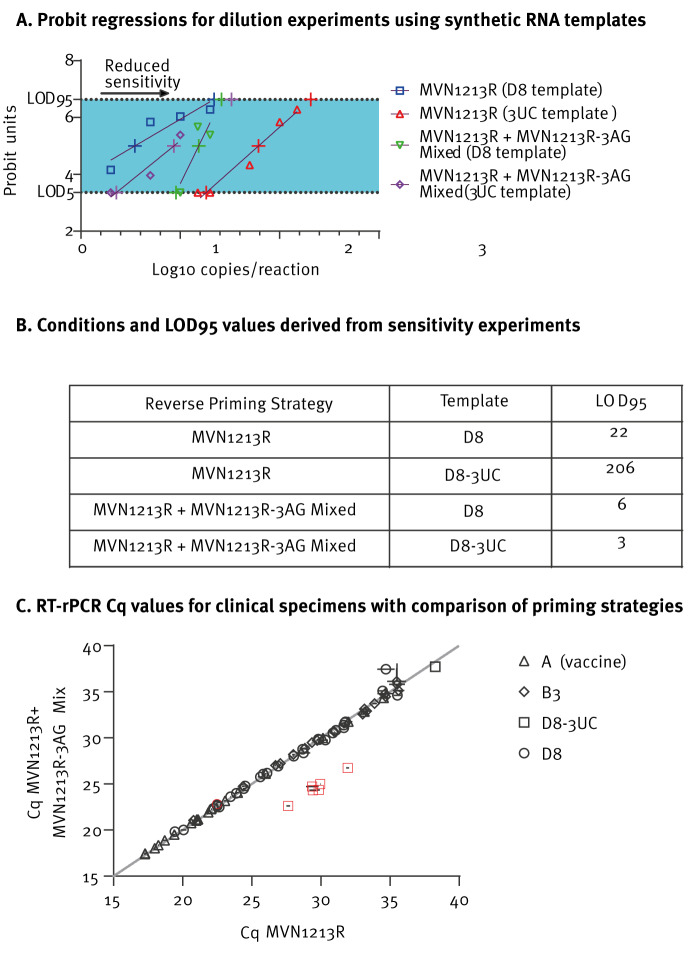
Summary of measles virus RT-rPCR sensitivities using synthetic RNA templates and clinical specimens

To investigate changes in assay sensitivity attributable to 3UC variant, we redesigned a reverse primer MVN1213R-3AG containing three compensatory substitutions to adequately bind 3UC (Figure 1B). We prepared two synthetic RNA constructs from MeV nucleoprotein sequences; the first was derived from sequence MVs/Illinois.US/20.16 [D8] and contained primer binding regions exactly matching the CDC assay. A 3UC variant template was derived from sequence MVs/Florida.US/37.23 [D8]. We provide additional detail on synthetic template preparation and related experimental conditions in the Supplement. Dilution series were prepared from the templates using 20 replicates per concentration and then reacted using MVN1213R alone or mixed with MVN1213R-3AG in equimolar ratios, a total of four conditions. Log_10_-transformed reaction copy numbers were regressed against probit-transformed positive rates using discernible linear portions of the assay response, extrapolating for limit of detection (LOD_95_). Fitted LOD_95_ values demonstrated reduced sensitivity for MVN1213R against the 3UC template (LOD_95_ = 206 copies/reaction) vs the baseline D8 sequence (LOD_95_ = 22 copies/reaction). Sensitivity to 3UC was rescued when using the mix of reverse primers (LOD_95_ = 33 copies/reaction) (Figure 2A). For each unique sequence in our surveillance dataset, we calculated the disassociation constant K_D_ for all binding combinations of primers or probe; our estimates of binding energetics (3UC + MVN1213R: K_D_ = 1.24 × 10^−5^_,_ 3UC + MVN1213R-3AG: K_D_ = 2.05 × 10^−12^) support improved sensitivity of MVN1213R-3AG to 3UC variant sequences. Methods for thermodynamic calculations are described in the Supplement. 

Cq: quantification cycle; LOD: limit of detection.

Panel A: Regression of probit-transformed hit rates along linear response range for combinations of priming strategy (50:50 mixed) with synthetic templates (D8 or D8–3UC). Fitted sensitivities LOD_5_, LOD_50_, and LOD_95_ (+) are depicted. Rightward shift along x-axis shows loss of sensitivity for combination of MVN1213R with 3UC mismatch template, with subsequent rescue of sensitivity when redesigned primer MVN1213R-3AG is added in equimolar concentration.

Panel C: Comparative RT-rPCR Cq values for clinical specimens show stability of response for both current (MVN1213R) and mixed (MVN1213R + MVN1213R-3AG) priming strategies. Grey: normal line; red: significant difference by Welch’s t-test (p < 0.05). Standard deviation (bars) is shown for both axes.

## Assay sensitivity using clinical specimens

Finally, we tested comparative performance of the mixed-priming design using clinical specimens (n = 83) obtained from CDC archive material. We selected specimens with a range of RNA loads to represent genotypes B3, D8 and A to ensure that the modified assay detected RNA from vaccine reactions (Table 2). Genotype D8 specimens represented sequences matching both MVN1213R and 3UC variants. Nucleic acid extracts were first reacted in triplicate with the CDC assay using MVN1213R only, then using the MVN1213R/MVN1213R-3AG mixed priming strategy. There were negligible significant differences in assay Cq across the two priming conditions, with statistically significant improvements of Cq observed only for 3UC variant specimens; this was attributable to both improved sequence complementarity and the increased GC content of the MVN1213R-AG binding duplex (Figure 2B). Extensive descriptions of experimental methods, with additional results and technical information are attached in the Supplement.

**Table 2:** Clinical measles virus specimens tested, with information on genotype and Cq range using MVN1213R priming, 2024 (n = 83)

Genotype	n	Mean Cq range using MVN1213R
A (vaccine)	20	20.78–35.61
B3	19	17.27–34.55
D8	30	19.43–35.54
D8–3UC	7	27.64–38.27
Negative	7	NA
**Total**	**83**	**17.27–38.27**

Cq: quantification cycle; NA: not applicable

## Discussion

We observed a detectable reduction in sensitivity for the MeV CDC assay when tested against a synthetic template bearing the 3UC variant sequence; the reduction in sensitivity was ameliorated by reverse priming redesign. Assay performance with clinical specimens was similar between current and redesigned priming strategies, with a relative decrease in 3UC specimen Cq values when priming with MVN1213R-3AG. 

Some limitations are noted: While the extent of 3UC variant circulation is described here using authoritative molecular data, N450 surveillance is subject to geographical and temporal biases; 3UC variant sequences remain relatively rare in the WHO database. Other priming mismatch patterns are observed in surveillance data; however, this study focused only on 3UC for reasons of recency, apparent prevalence in recent molecular surveillance data, and substitution multiplicity. While assay sensitivities remained high for all experimental conditions reported here, greater replicate allocation would offer better resolution of linear assay response ranges; sensitivity experiments were designed to balance accuracy against perceived urgency. We do not attempt here to speculate on shifts in false negative rates for the assay when 3UC variant sequences would be encountered in testing. However, the use of multitarget assays, while more technically complex, would in some cases provide useful data on priming destabilisation should it occur [6]. Our estimations of thermodynamic parameters used assumed salt concentrations and should be interpreted in a relative context only. MeV is noted for low rates of nucleotide substitution [7,8], explaining the stability of performance of the CDC assay since it was originally introduced in 2006 [1]. 

## Conclusion 

Despite the evident stability of the primer binding sites in circulating viruses, in silico monitoring of priming stability should be continually performed across molecular diagnostics interests to ensure stable performance for assays of this type. Laboratories using the CDC assay should consider introducing the new reverse priming strategy described here.

## Supplementary Data

382000 Supplement
