# Supplementary material for: Evaluation of the sensitivity of a measles diagnostic real-time RT-PCR assay incorporating recently observed priming mismatch variants, 2024
Source: Euro Surveill. 2024 Jul 11;29(28):2400410. doi: 10.2807/1560-7917.ES.2024.29.28.2400410 (PMC11241853; doi:10.2807/1560-7917.ES.2024.29.28.2400410)
Supplement: Supplement [file 24-00410_BECK_Supplement.pdf]

SUPPLEMENTAL INFORMATION

TITLE

Rapid Communication: Evaluation of the Sensitivity of a Measles Diagnostic Real-Time RT-PCR Assay Incorporating Recently Observed Priming Mismatch Variants

DISCLAIMER

This supplementary material is hosted by *Eurosurveillance* as supporting information alongside the article *Evaluation of the sensitivity of a measles diagnostic real-time RT-PCR assay incorporating recently observed priming mismatch variants, 2024*, on behalf of the authors, who remain responsible for the accuracy and appropriateness of the content. The same standards for ethics, copyright, attributions and permissions as for the article apply. Supplements are not edited by *Eurosurveillance* and the journal is not responsible for the maintenance of any links or email addresses provided therein.

TABLE OF CONTENTS

SUPPLEMENTAL INFORMATION 1
TABLE OF CONTENTS 1
METHODS 2
Clinical Specimens 2
Production of Synthetic RNA Constructs For Sensitivity Comparisons 2
Genotype D8 (no-mismatch baseline) and 3UC (3 mismatch variant) Synthetic Templates 2
RT-rPCR Assay at Baseline (CDC Assay) 3
Baseline Assay Conditions, Analysis of Clinical Specimens 3
Specimen Controls 3
Plate Controls 3
Interpretation 3
RT-rPCR Assay with Reverse Primer Modifications 4
Sensitivity Experiments 4
Genotype D8 (no-mismatch baseline) and 3UC (3 mismatch variant) Synthetic Templates 4
Dilution Experiments with Synthetic RNA Templates 4
Probit Analysis of Dilution Experiments 4
Clinical Specimens 5
In Silico Analyses and Epidemiologic Data 5

Source Data 5
Primer Mismatch Identification 5
Thermodynamic Calculations 5
FIGURES 7
Figure S1: Comparison of C_t values for clinical specimens, genotype D8 (no priming mismatch). 7
Figure S2: Comparison of C_t values for clinical specimens, genotype D8 (3UC reverse priming mismatch). 8
Figure S3: Comparison of C_t values for clinical specimens, genotype B3 (no priming mismatch). 9
Figure S4: Comparison of C_t values for clinical specimens, genotype A (Vaccine strain, no priming mismatch). 10
Figure S5: Mismatch variants observed for CDC assay forward primer MVN1139F. 11
Figure S6: Mismatch variants observed for CDC assay reverse primer MVN1213R. 12
Figure S7: Mismatch variants observed for CDC assay probe. 13
SUPPLEMENTARY REFERENCES 13

METHODS

Clinical Specimens

Clinical specimens were received as nasopharyngeal or throat swabs in viral transport media (VTM), or as urine. Viral RNA was extracted from VTM or urine using the Qiagen Viral RNA Mini Kit (Germantown MD). Swab eluates and all extracted vRNAs were stored at -80°C until tested.

Production of Synthetic RNA Constructs For Sensitivity Comparisons

Genotype D8 (no-mismatch baseline) and 3UC (3 mismatch variant) Synthetic Templates

A genotype D8 synthetic RNA was derived from MeV specimen MVs/Illinois.USA/20.16 [D8] and produced by RT-PCR directly from clinical specimen material. A 3UC synthetic RNA template was derived from MeV specimen MVs/Florida.USA/37.23[D8] and produced by RT-PCR directly from clinical specimen material. For both templates, 5uL of extracted RNA was reacted with primers T7NF-5`-TAATACGACTCACTATAGGGCAGGATTAGGGTAATCCGAG-3` and MeV214-5`-TAACAATGATGGAGGGTAGG-3`(with final concentration 200nM), yielding a whole MeV nucleoprotein with an upstream T7 promoter element. Reaction conditions were as follows, using Invitrogen Superscript III One-step RT-PCR kit (Thermo Fisher): 55°C for 30min, followed by 40 Cycles of 95°C/15sec, 55°C/30sec, 68°C/30sec , then holding at 68°C/5min. The amplicon was cleaned using the ChargeSwitch-Pro Cleanup kit (Invitrogen/Thermo Fisher). 100ng of amplicon DNA was transcribed using the MEGAscript T7 Transcription kit (Thermo Fisher). Copy numbers for sensitivity tests were calculated using a nominal length of 1651 bases.

RT-rPCR Assay at Baseline (CDC Assay)

This section describes typical use of the CDC assay for analyses of clinical specimens as is reported here. Reaction conditions/program are identical for all experiments described below. Controls are modified or removed for some experiments when not indicated.

Baseline Assay Conditions, Analysis of Clinical Specimens

The CDC assay was performed as originally described by Hummel(1), with modifications. Specimens were reacted in 20µL reaction volume, in duplicate wells, using an ABI 7500 Fast Dx Real-Time PCR System (Applied Biosystems, CA). Master mixes were prepared using Quantitect® RT-PCR reagents (Qiagen, Germantown, MD). Program conditions were as follows: Reverse transcription at 50°C for 20min, Denaturation/activation at 95°C for 15min, then 40 cycles of denaturation at 95°C for 5 seconds, followed by anneal/extend at 60°C for 1min. Fluorescence was captured on anneal/extend segments. MeV primers were added to a final concentration of 300nM. MeV probe (Table S1) was added to a final concentration of 250nM. 2µL of specimen (synthetic template or specimen extract) were added to the reaction mix. Dilutions of synthetic template were performed in TETX (0.1% Triton X-100 in Tris-EDTA, pH 7.0). Thresholding was manually adjusted in reference to positive control wells (Plate Controls, below), considering both (1) historically acceptable response ranges and (2) divergence of duplicate wells at exponential phase of amplification.

Specimen Controls

For all clinical specimens, a human RNAseP target was reacted in separate wells. Primers were added to a final concentration of 300nM. Primers for RNAseP reaction are shown in Table S1.

Plate Controls

Plates controls were prepared in duplicate wells. (1) Positive control template was derived from genotype D8 specimen MVs/Illinois.USA/20.16 (2×10^5 and 2×10^3 copies/reaction) and diluted in TETX (0.1% Triton X-100 in Tris-EDTA, pH 7.0). (2) No-template control (NTC) was prepared with 2µL of molecular grade water. (3) Positive/Negative extraction controls were analyzed upon initial sample extraction and are not used here.

Interpretation

For clinical specimen experiments, mean duplicate C_t values were obtained in triplicate for statistical comparison of priming strategies. For dilution/probit experiments (below), positive hit rates were determined using a C_t cutoff of 38.

Table S1: Primers and Probes used in CDC assay and sensitivity studies (Repeated here and in main text)

Oligonucleotide	Sequence
**Measles Virus Nucleoprotein**	
Forward Primer (MVN1139F)	5′ TGGCATCTGAACTCGGTATCAC 3′

Reverse Primer (MVN1213R)	5′ TGTCCTCAGTAGTATGCATTGCAA 3′
Probe	5′ FAM-CCGAGGATGCAAGGCTTGTTTCAGA-BHQ 3′
Modified Reverse Primer (MVN1213R-3AG)	5′ TGTCCTCGGTGGTGTGCATTGCAA 3′<br>[Modified bases from MVN1213R are highlighted]
**Human RNaseP**	
HURNASE-P-F	5′ AGATTTGGACCTGCGAGCG 3′
HURNASE-P-R	5′ GAGCGGCTGTCTCCACAAGT 3′
HURNASE-P-P	5′ FAM-TTCTGACCTGAAGGCTCTGCGCG-BHQ 3′

RT-rPCR Assay with Reverse Primer Modifications

For all experiments using reverse primer modifications, the redesigned reverse primer MVN1213-3AG was added in a 50:50 molar ratio with the unmodified primer the MVN1213R during master mix preparation, for a final concentration of 150nM/primer.

Sensitivity Experiments

Genotype D8 (no-mismatch baseline) and 3UC (3 mismatch variant) Synthetic Templates

A genotype D8 synthetic RNA was derived from MeV specimen MVs/Illinois.USA/20.16 [D8] and produced by RT-PCR directly from clinical specimen material. A 3UC synthetic RNA template was derived from MeV specimen MVs/Florida.USA/37.23[D8] and produced by RT-PCR directly from clinical specimen material. For both templates, 5µL of extracted RNA was reacted with primers T7NF-5`-TAATACGACTCACTATAGGGCAGGATTAGGGTAATCCGAG-3` and MeV214-5`-TAACAATGATGGAGGGTAGG-3`(with final concentration 200nM), yielding a whole MeV nucleoprotein with an upstream T7 promoter element. Reaction conditions were as follows, using Invitrogen Superscript III One-step RT-PCR kit (Thermo Fisher): 55°C for 30min, followed by 40 Cycles of 95°C/15sec, 55°C/30sec, 68°C/30sec , then holding at 68°C/5min. The amplicon was cleaned using the ChargeSwitch-Pro Cleanup kit (Invitrogen/Thermo Fisher). 100ng of amplicon DNA was transcribed using the MEGAscript T7 Transcription kit (Thermo Fisher). Copy numbers for sensitivity tests were calculated using a nominal length of 1651 bases.

Dilution Experiments with Synthetic RNA Templates

Assay sensitivity was determined using D8/D8-3UC synthetic templates. Dilution series were prepared from templates along a range of 10^6 – 1copy/µL in TETX. 2µL of diluted template was reacted using assay conditions as previously described (above). Probit analysis (below) was performed on positive hit rates (C_t <38) derived from the dilution series reactions to derive limits of detection.

Probit Analysis of Dilution Experiments

Hit rates were calculated as the proportion of positive (C_t <38) wells (N=20) for each combination of template dilution and priming condition. Rates were converted to Probit units using the NORMSINV() function in Microsoft Excel v.2308, adding a Y-offset of 5. Log10-converted copy numbers were regressed against probit-transformed hit rates using the Simple Linear Regression function in Graphpad v.10.2. Regressions were

hand-adjusted to accommodate the linear response range of the dilution series, using a minimum of three points for each combination of template and priming strategy. Limits of detection were derived from fitted curves (LOD_{95}=6.64, LOD_{50}=5.00, LOD_{5}=3.36), and expressed as template copies per reaction. In all experimental conditions, assay linear response occurred at or below 206 copies/reaction; sensitivity was high even for 3UC priming mismatch conditions.

Clinical Specimens
For all clinical specimens, 2µL of RNA extract was reacted in duplicate wells (triplicate, three pairs of wells total) using conditions described above. Each specimen extract was reacted twice, under the following conditions: (A) Using the reverse priming strategy as described by Hummel (1) [MVN1213R] and (B) using an equimolar mixture of reverse primers [MVN1213R + MVN1213R-3AG]. C_t values were compared for the priming conditions using Welch's t-test as implemented in Graphpad v.10.2. Summary of comparisons for priming conditions are shown in Figures S1-S4.

In Silico Analyses and Epidemiologic Data

Source Data
With permission, a dataset was obtained containing all MeV genotype D8 and B3 sequences deposited to MeaNS between 2003 and 2023, inclusive. Sequences were deduplicated to obtain a set of unique DSIds; this filtered set was used for primer/probe alignment tasks and mismatch quantification. Tables representing oligonucleotide mismatch counts were joined to the original submission table to obtain quantifications of primer/probe mismatch with respect to individual submissions and within aggregated quarterly time blocks. All data processing was performed using common table operations in Pandas v.1.4.3 (Python v. 3.9.13), hosted on JupyterLab v.3.4.5.

Primer Mismatch Identification
Primer mismatches were identified by performing a global pairwise alignment for each primer-target sequence pair. Alignments were calculated using the Needleman-Wunsch algorithm as implemented in Biopython v1.83. To eliminate gaps in the alignment, gaps were assigned a −10 penalty in the alignment scoring scheme, while matches and mismatches were assigned 1 and 0 points respectively. Temporal patterns for all detected mismatches are shown [Figures S5,6,7]. This analysis considers oligonucleotides independently; information is not shown here for linked mismatch patterns that may occur across multiple binding regions in the same template. Mismatch patterns represent a minority of sequences in the complete dataset of genotype B3 or D8 submissions.

Thermodynamic Calculations
The Gibbs free energy difference ΔG for primer-target formation was calculated using the nearest-neighbor model and thermodynamic parameters originally published by Allawi and SantaLucia (2). The temperature T was set to match the PCR protocol annealing temperature (60° C). The monovalent salt correction term was determined using typical PCR buffer conditions (20 mM Tris, 50 KCl, 4 mM MgCl2, 200 $\mu\mathrm{M}$ dNTP) (3, 4). The dissociation constant K_D was then calculated with ΔG using the formula:

$$K_D = \exp\left(\frac{\Delta G}{RT}\right)$$

K_D was estimated for all combinations of distinct N450 sequences with primer(s) and probe.

FIGURES

Figure S1: Comparison of C_t values for clinical specimens, genotype D8 (no priming mismatch).

Specimens (n=30) were reacted in triplicate using reverse priming strategies MVN1213R and MVN1213R+MVN1213R-3AG. Mean C_t for priming strategies was compared using Welch's t-test. Significant difference was observed for one of 30 specimens. *p<0.05

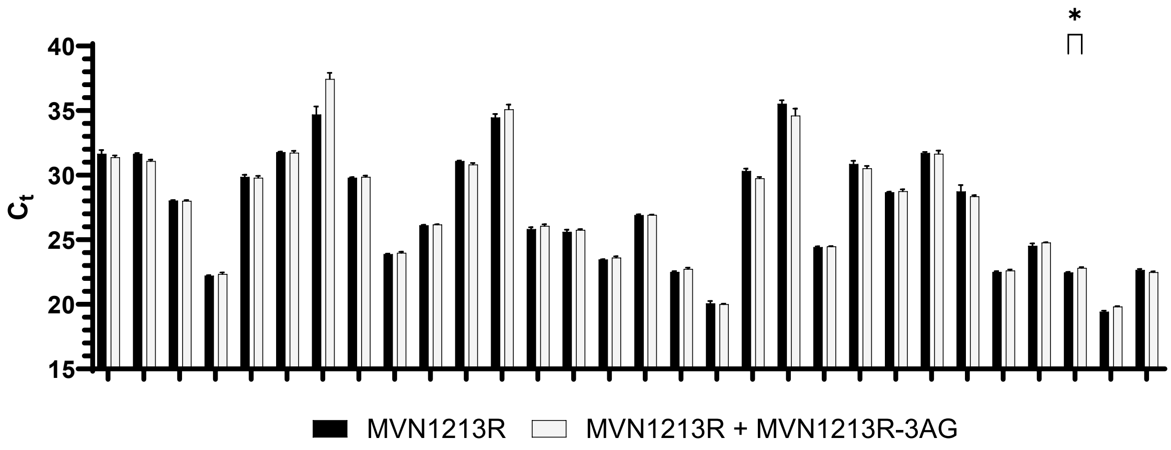

Figure S2: Comparison of C_t values for clinical specimens, genotype D8 (3UC reverse priming mismatch).

Specimens (n=7) were reacted in triplicate using reverse priming strategies MVN1213R and MVN1213R+MVN1213R-3AG. Mean C_t for priming strategies were compared using Welch's t-test. Significant difference of C_t was observed for 6 of 7 specimens; in these cases, lower C_t (increased sensitivity) was observed for the mixed priming strategy. For one reaction pair (+), two of three triplicate wells were undetected when using MVN1213R, and so statistical comparison was not possible; three wells were detected when using the mixed priming strategy. The number of 3UC specimens was limited to those available in the US. *p<0.05

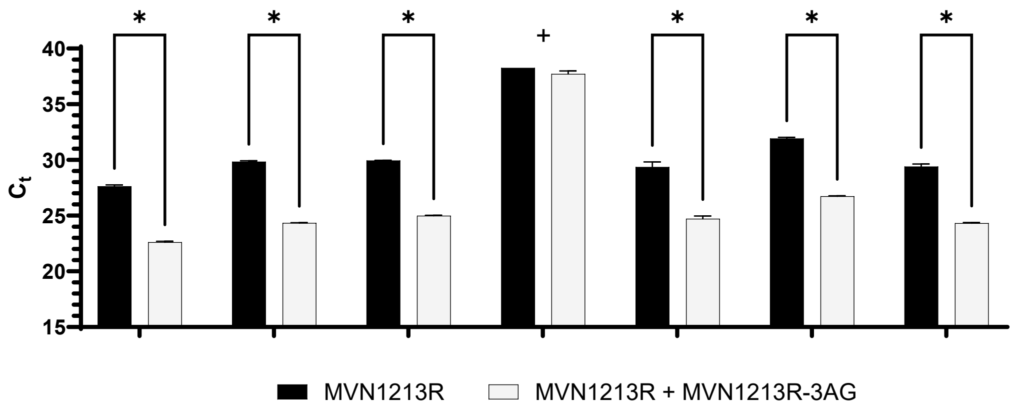

Figure S3: Comparison of C_t values for clinical specimens, genotype B3 (no priming mismatch).

Specimens (n=19) were reacted in triplicate using reverse priming strategies MVN1213R and MVN1213R+MVN1213R-3AG. Mean C_t for priming strategies were compared using Welch's t-test. No significant differences were observed. *p<0.05

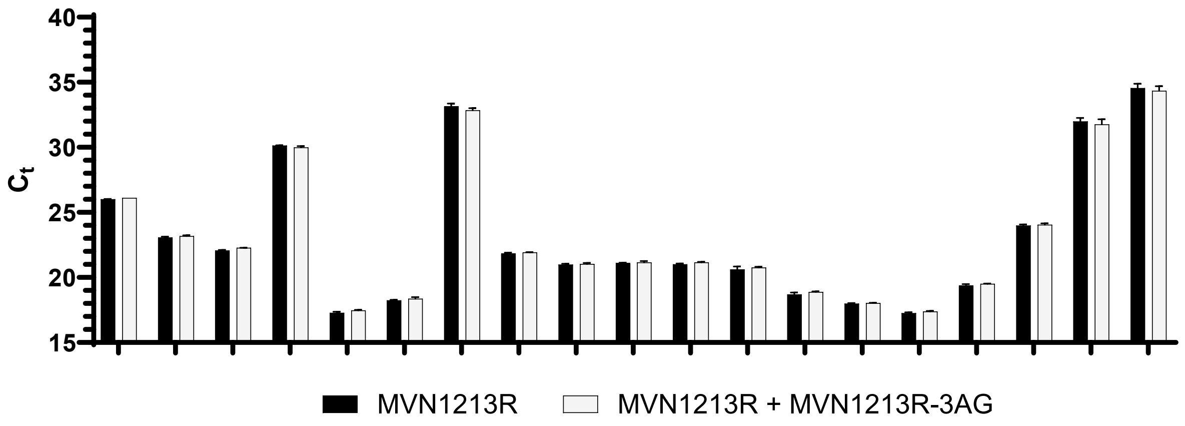

Figure S4: Comparison of C_t values for clinical specimens, genotype A (Vaccine strain, no priming mismatch).

Specimens (n=20) were reacted in triplicate using reverse priming strategies MVN1213R and MVN1213R+MVN1213R-3AG. Mean Ct for priming strategies were compared using Welch's t-test. No significant differences were observed. *p<0.05

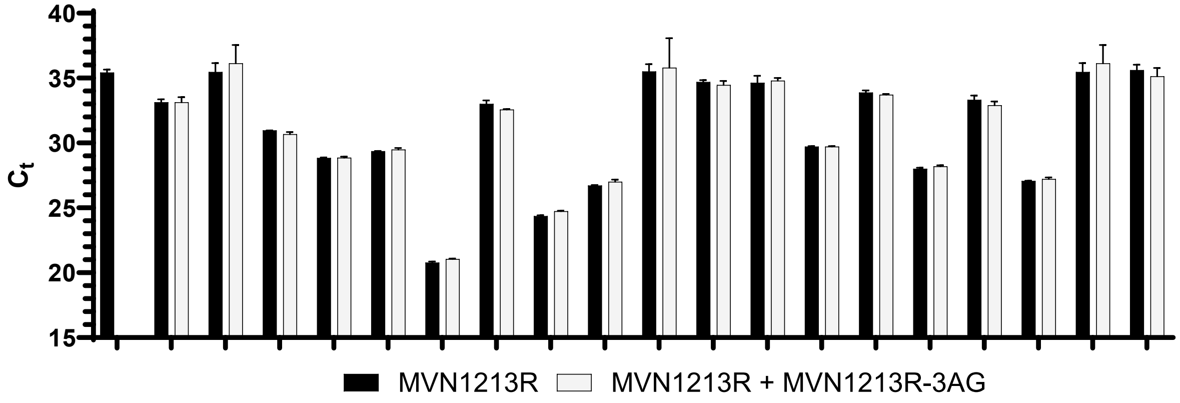

Figure S5: Mismatch variants observed for CDC assay forward primer MVN1139F.

Temporal distribution of MeaNS N450 submissions is shown with quarterly binning (end date of quarter shown).

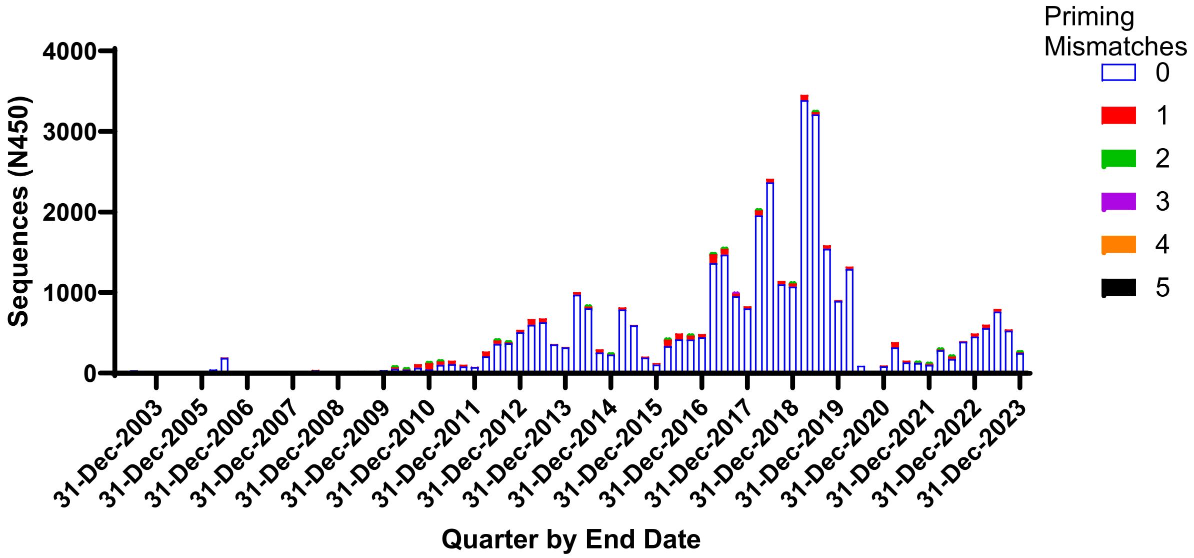

Figure S6: Mismatch variants observed for CDC assay reverse primer MVN1213R.

Temporal distribution of MeaNS N450 submissions is shown with quarterly binning (end date of quarter shown).

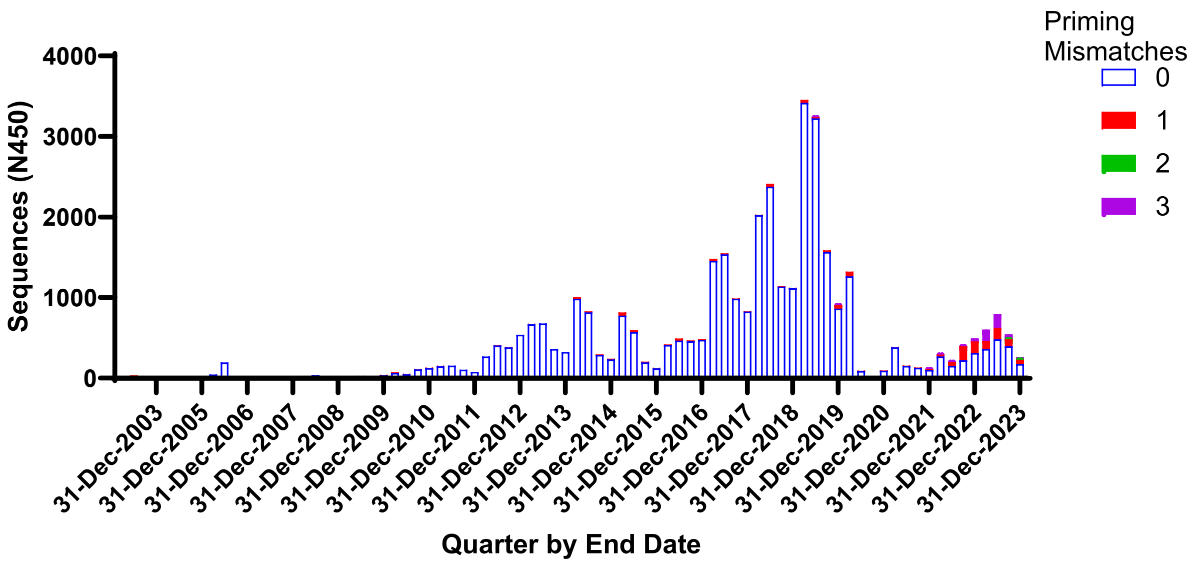

Figure S7: Mismatch variants observed for CDC assay probe.
Temporal distribution of MeaNS N450 submissions is shown with quarterly binning (end date of quarter shown).

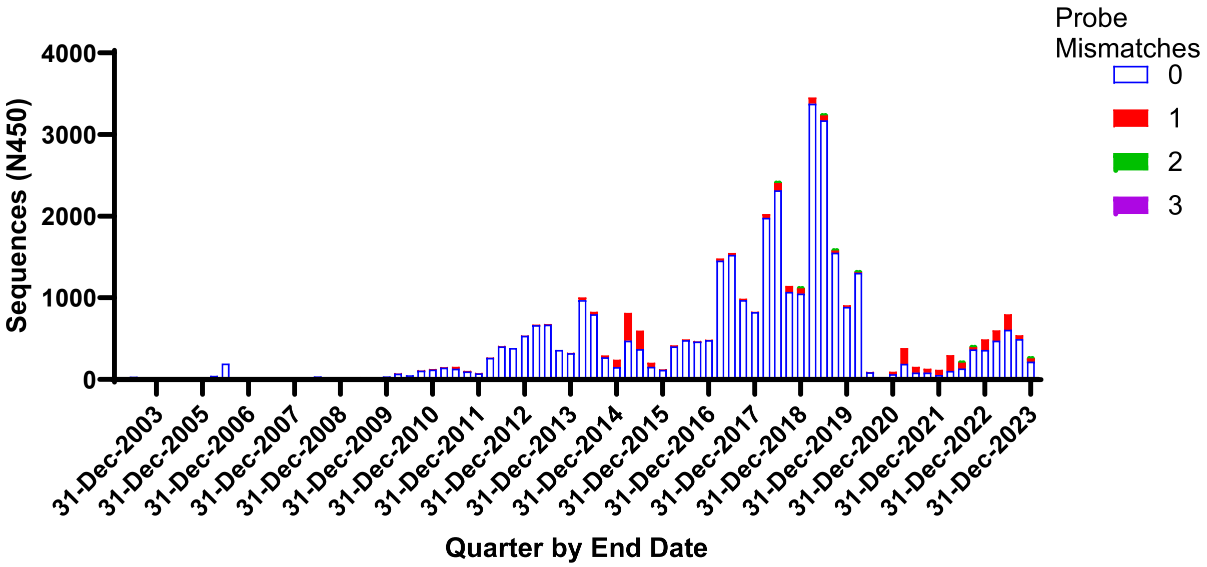

SUPPLEMENTARY REFERENCES

1. Hummel KB, Lowe L, Bellini WJ, Rota PA. Development of quantitative gene-specific real-time RT-PCR assays for the detection of measles virus in clinical specimens. J Virol Methods. 2006;132(1-2):166-73.
2. Allawi HT, SantaLucia J, Jr. Thermodynamics and NMR of internal G.T mismatches in DNA. Biochemistry. 1997;36(34):10581-94.
3. von Ahsen N, Wittwer CT, Schutz E. Oligonucleotide melting temperatures under PCR conditions: nearest-neighbor corrections for Mg(2+), deoxynucleotide triphosphate, and dimethyl sulfoxide concentrations with comparison to alternative empirical formulas. Clin Chem. 2001;47(11):1956-61.
4. SantaLucia J, Jr. A unified view of polymer, dumbbell, and oligonucleotide DNA nearest-neighbor thermodynamics. Proc Natl Acad Sci U S A. 1998;95(4):1460-5.
